# Low Bone Mineral Density on Computed Tomography: Association with Poor Survival after Transcatheter Aortic Valve Replacement

**DOI:** 10.3390/jcm13092698

**Published:** 2024-05-03

**Authors:** Caglayan Demirel, Kseniya Halavina, Kevin Hamzaraj, Johanna Klement, Manar El-Shaer, Rayyan Hemetsberger, Max Paul Winter, Sophia Koschatko, Charlotte Jantsch, Martin Andreas, Christian Loewe, Andreas Kammerlander, Christian Hengstenberg, Philipp E. Bartko

**Affiliations:** 1 Department of Internal Medicine II, Clinical Division of Cardiology, Medical University of Vienna, 1090 Vienna, Austria; caglayan.demirel@meduniwien.ac.at (C.D.); kseniya.halavina@meduniwien.ac.at (K.H.); kevin.hamzaraj@meduniwien.ac.at (K.H.); n11916429@students.meduniwien.ac.at (J.K.); n11844296@students.meduniwien.ac.at (M.E.-S.); rayyan.hemetsberger@meduniwien.ac.at (R.H.); max-paul.winter@meduniwien.ac.at (M.P.W.); sophia.koschatko@meduniwien.ac.at (S.K.); andreas.kammerlander@meduniwien.ac.at (A.K.); christian.hengstenberg@meduniwien.ac.at (C.H.); 2 Department of Internal Medicine II, Clinical Division of Cardiac Surgery, Medical University of Vienna, 1090 Vienna, Austria; martin.andreas@meduniwien.ac.at; 3 Department of Radiology, Medical University of Vienna, 1090 Vienna, Austria; christian.loewe@meduniwien.ac.at

**Keywords:** aortic valve stenosis, bone mineral density, computed tomography, mortality

## Abstract

**Background:** Transcatheter aortic valve replacement (TAVR) has evolved as first-line therapy for severe aortic valve stenosis (AS), with pre-procedural computed tomography (CT) providing critical anatomical information. While primarily used for anatomical planning, TAVR-CT also offers an opportunity to assess low bone mineral density (BMD), a known indicator of frailty. Despite this, the prognostic role of BMD in TAVR patients remains unknown. This study aimed to evaluate BMD on routine TAVR-CT and its impact on long-term survival. **Methods:** In this retrospective study, 770 consecutive TAVR patients (mean age 80.7 ± 6.7 years, 54.0% males) between November 2015 and March 2022 were included. BMD was measured from a single axial image at the thoracic vertebral level on unenhanced CT scans. Cox regression models assessed the impact of BMD on mortality, and Restricted Cubic Spline models identified potential mortality thresholds. **Results:** The mean BMD value, as measured on non-contrast CT, was 147.5 ± 5.4 Hounsfield units, demonstrating a noteworthy association with mortality (adjusted hazard ratio per 100 HU decrease: 1.27 [95%CI: 1.01–1.59], *p* = 0.041). Restricted cubic spline analysis indicated that BMD below 200 HU was linked to a substantial increase in mortality risk. Upon crude Cox regression analysis, every 100 HU decrease was associated with a 32% increase in risk for death (HR 1.32 [95%CI: 1.068–1.65)], *p* = 0.010). **Conclusions:** In conclusion, low BMD on TAVR-CT is independently associated with reduced survival, suggesting its potential as a tool for comprehensive frailty assessment and improved risk prediction in TAVR patients.

## 1. Introduction

Transcatheter aortic valve replacement (TAVR) has emerged as a transformative therapeutic modality for individuals afflicted with severe aortic valve stenosis (AS), heralding a new era in cardiovascular medicine. Over the past two decades, TAVR has undergone remarkable evolution, marked by substantial enhancements in safety, efficacy, and procedural outcomes. These advancements led to a notable rise in the utilization of TAVR procedures, encompassing a broader spectrum of patients, including those deemed to be at intermediate or low surgical risk [1,2,3,4,5,6,7].

The paradigm shift brought about by TAVR is underscored by a comprehensive analysis of individual patient data gleaned from randomized clinical trials and meta-analyses. These studies furnish compelling evidence of TAVR’s superiority over surgical aortic valve replacement (SAVR), particularly in terms of clinical endpoints such as mortality, disabling stroke, and composite outcomes. Notably, the one-year evaluation reveals a tangible advantage for TAVR, characterized by a reduced risk profile compared to SAVR, thus positioning it as a compelling alternative [5].

Delving deeper into the landscape of clinical outcomes, longitudinal studies have provided further insights into the comparative efficacy of TAVR and SAVR, particularly among patients with severe aortic stenosis and intermediate surgical risk. These investigations suggest a remarkable parity in outcomes between the two modalities, with no discernible disparities in mortality or disabling stroke incidence even at the five-year mark [6]. 

Such findings underscore the robustness and durability of TAVR outcomes, bolstering its standing as a viable therapeutic option across diverse patient cohorts.

Moreover, extending the investigative lens to encompass low-risk patient populations has yielded illuminating insights into TAVR’s efficacy relative to surgery. Seminal studies have validated the non-inferiority of TAVR, particularly when employing self-expanding supraannular bioprostheses or balloon-expandable valves. These investigations, focusing on critical endpoints such as death or disabling stroke, reaffirm TAVR’s efficacy and durability, positioning it as a credible alternative to SAVR [1,2]. 

The collective body of evidence underpins TAVR’s favorable outcomes across a spectrum of risk profiles, cementing its status as the cornerstone of contemporary cardiovascular care. Its superior clinical efficacy, coupled with its minimally invasive nature, renders TAVR an attractive therapeutic option for patients and clinicians alike. Moreover, the burgeoning utilization of TAVR procedures underscores its pivotal role in addressing the unmet clinical needs of patients with severe aortic stenosis, particularly those deemed to be at high risk for conventional surgical interventions.

In a contemporary context, the observed variability in the incidence rates of TAVR and SAVR procedures across different healthcare settings underscores the multifactorial nature of procedural utilization.

In the evolving landscape of TAVR, the significance of a thorough pre-procedural assessment cannot be overstated. While traditional evaluations focus on anatomical parameters like access size and valve characteristics, there is a growing acknowledgment of the need to integrate broader health metrics into the preparatory phase. A comprehensive evaluation that considers patient comorbidities, overall medical condition, and frailty parameters is essential for optimizing patient selection and tailoring treatment strategies to individual needs.

However, estimating frailty poses a significant challenge in clinical practice due to its multifactorial nature, which encompasses various aspects of vulnerability, including nutritional status, physical and cognitive impairments, and laboratory values [8,9].

This complexity underscores the need for a comprehensive assessment approach that considers the diverse factors contributing to frailty.

In the pursuit of objective frailty markers, prior investigations have delved into the analysis of body composition parameters, leveraging the opportunistic utilization of pre-TAVR-CT scans, with a particular focus on two pivotal tissues: muscle mass and fat mass. This investigative approach extends beyond conventional cardiac risk assessment paradigms to encompass a broader spectrum, inclusive of noncardiac risk assessment considerations. The exploration of muscle mass and fat mass as objective markers of frailty introduces a comprehensive dimension to risk assessment protocols [10,11,12,13,14,15,16]. Traditionally, frailty assessments have predominantly revolved around physiological and functional domains. However, the incorporation of body composition parameters adds a more tangible and quantifiable aspect to the evaluation process. This methodology acknowledges the systemic nature of frailty, recognizing that alterations in body composition can exert profound implications for overall health and resilience. The inclusion of muscle mass as a parameter holds particular relevance, as it not only reflects an individual’s physical strength but also signifies their capacity to endure stressors and recuperate from interventions.

Further, the evaluation of fat mass acknowledges the intricate role of adipose tissue in metabolic and inflammatory processes, thereby offering insights into the overall metabolic health of the patient [17,18,19,20]. 

In a prior study by Demirel et al., various body composition parameters were examined to comprehensively assess frailty using obligatory CT scans prior to TAVR. Volumetric assessment of each CT scan was conducted on a single axial image obtained at the level of the middle third lumbar vertebra (L3), focusing on the analysis of visceral adipose tissue (VAT) and total muscle area (TMA). This investigation highlighted that a reduced total muscle area was linked to elevated 5-year all-cause mortality, presenting an additional metric for frailty assessment. Moreover, the impact of low TMA on 5-year all-cause mortality was contingent upon sex. Specifically, a protective effect associated with higher TMA levels was discerned in male patients (pinteraction: sex × TMA = 0.007). Conversely, appendicular muscle area (PMA) and VAT were not correlated with increased 5-year mortality. Furthermore, the combination of TMA and VAT did not exhibit enhanced predictive power for lower survival rates [7]. 

By expanding the analysis of body composition parameters to encompass noncardiac risk assessment, these studies broaden the scope of frailty evaluation. This holistic approach underscores the imperative of considering multifaceted determinants of frailty, thereby facilitating more informed clinical decision making and comprehensive patient care in the context of transcatheter aortic valve replacement.

However, despite these advancements, the role of low bone mineral density (BMD), which could also be measured on pre-TAVR-CT scans, remains unclear, although it is recognized as a predictor of frailty observed across various medical conditions.

Research has consistently demonstrated a robust association between low BMD and an elevated risk of cardiovascular events, highlighting the intricate relationship between musculoskeletal health and overall cardiovascular health [21,22]. For instance, individuals with lower BMD have been shown to be at an increased risk of mortality, particularly among male populations [23]. Additionally, adults with complex congenital heart disease often exhibit lower total BMD compared to their healthy counterparts [24].

The association between BMD and outcomes in patients undergoing TAVR represents an intriguing avenue of investigation. While the prognostic significance of low BMD in various medical conditions is well established, its impact on TAVR outcomes remains less explored. Osteoporosis, characterized by low BMD, is known to increase the risk of fractures in adults [25]. OP and osteoporotic fractures are associated with disability, impaired quality of life, and mortality [26,27]. Studies consistently highlight the association between low BMD and heightened mortality risk, underscoring the importance of considering bone health in clinical decision making [22,28].

Also, multimorbidity is common in individuals with osteoporosis, and early identification not only allows for the implementation of treatment strategies but also presents an opportunity to improve decision making in frail patients [27]. Furthermore, the economic ramifications of osteoporosis-related complications emphasize the need for effective risk assessment strategies. Previous studies observed that the impact of OP is even more noticeable when considering the cost factors for the U.S. healthcare system, with approximately USD 17 billion annually, with an annual cost projected to approach USD 50 billion by the year 2040 [29].

Integrating BMD assessment into the pre-TAVR evaluation process holds promise for enhancing risk stratification and optimizing patient management. Prior research has demonstrated the feasibility of opportunistically measuring BMD using routine abdominal CT scans, offering a cost-effective and radiation-minimized approach [30,31].

HU measurements taken from the C4 vertebral body were performed with a correlation of low BMD of the femoral neck as determined by DXA scan T-scores and revealing a high degree of sensitivity and negative predictive value of 80% [32]. Further, routine cardiac CT thoracic bone mineral density (BMD) was measured to identify individuals who have low BMD and a greater fracture rate [33].

Our study seeks to elucidate the predictive value of BMD in TAVR patients, particularly concerning frailty and mortality outcomes. We hypothesize that low BMD serves as a surrogate marker for frailty and is associated with increased all-cause mortality risk in this patient cohort. To investigate this hypothesis, we aimed to assess BMD opportunistically from pre-TAVR-CT scans and evaluate its feasibility and impact on procedural outcomes.

Through a comprehensive evaluation of BMD and its implications for frailty and mortality in TAVR patients, our research aims to contribute to personalized risk assessment strategies and optimize patient care pathways. By advancing our understanding of the interplay between bone health and TAVR outcomes, we aim to inform clinical practice guidelines and improve patient outcomes in the context of transcatheter interventions for aortic valve replacement.

## 2. Materials and Methods

### 2.1. Study Population

The Vienna TAVR registry is a prospective registry enrolling consecutive patients undergoing TAVR performed by the Department for Cardiology and Cardiac Surgery, Vienna University Hospital. We included all patients treated with TAVR at Medical University Hospital Vienna (Department of Cardiology and Cardiac Surgery) between November 2015 and March 2022 (*n* = 1223). In 350 cases, only CT angiography scans were available. In 103 cases, CT scans were performed in referring centers and not available for postprocessing. Thus, the final cohort comprised 770 patients in whom non-contrast CT scans were available for BMD assessment. In the Supplementary section, we provide a descriptive analysis comparing included and excluded patients. This analysis aims to elucidate any differences between these groups. Notably, the percentage of missing variables for each variable is reported to be less than 5%, ensuring robustness in the analysis.

### 2.2. Transcatheter Aortic Valve Replacement

TAVR procedures, predominantly conducted via the transfemoral route, were executed in accordance with institutional protocols, utilizing either local or general anesthesia within a hybrid catheterization laboratory setting. As per established guidelines, pre-existing oral anticoagulation was intentionally halted prior to the TAVR procedure. During the intervention, patients were administered unfractionated heparin with a targeted activated clotting time of 250 s, adhering to predetermined protocols. Simultaneously, any ongoing antiplatelet therapy was continued based on the patient’s pre-existing regimen. This standardized approach reflects adherence to established best practices, prioritizing procedural precision and patient safety throughout the transcatheter aortic valve implantation process.

### 2.3. Study Design

Retrospective data collection with follow-ups was used with standardized case report forms (medical history, baseline clinical, procedural, and follow-up). For the purpose of the present study, the BMD measurement was performed for each patient receiving TAVR.

### 2.4. CT Assessment

**Figure 1:**
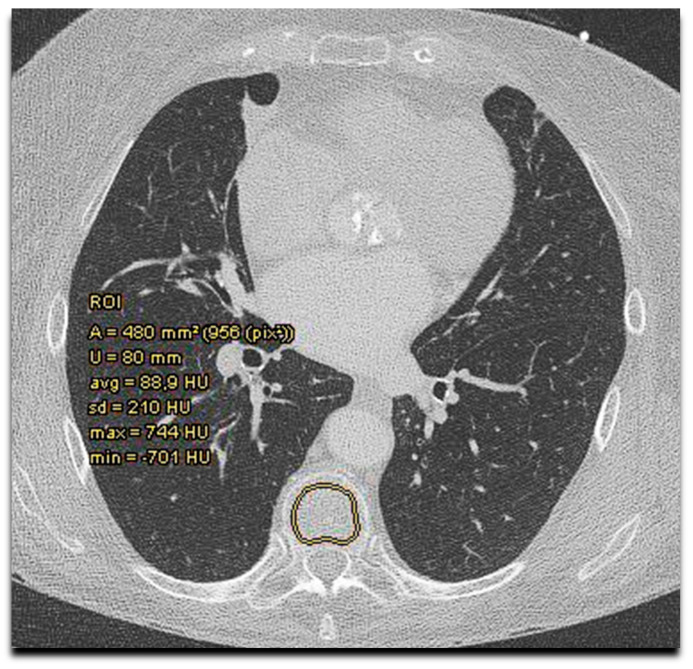
Bone mineral density assessment by Hounsfield units measurement on TAVR-CT scan. Bone mineral density (Hounsfield units) measurement in sternum and thoracal vertebra with determination of area (A), circumference (U), average (avg) density, standard deviation (SD), and maximal (max) and minimal (min) density in the region of interest (ROI).

The obligatory TAVR-CT angiography scans were performed according to standard protocols [34]. In addition, a non-contrast-enhanced acquisition (120 KV, 3 mm slices) covering the area from the aortic root to the apex included coronary and aortic valve calcium scoring (CAC, AVC). For bone density measurement, HU values of thoracal vertebrae on the non-contrast CT scan were assessed (Figure 1). This was performed by Deep Unity, a widely used picture archiving and communication system (Dedalus HealthCare, Konrad-Zuse-Platz 1–3, Bonn, Germany).

### 2.5. Data Collection

A combination of retrospective and prospective data collection methodologies was employed, accompanied by follow-up assessments utilizing standardized case report forms encompassing medical history, baseline clinical information, procedural details, and subsequent follow-up records.

The Vienna TAVR registry encompasses clinical follow-up evaluations at 30 days, 1 year, and 3 years post-TAVR. Survival data pertaining to all-cause mortality were regularly updated on an annual basis through the integration of information from the centralized death registry covering the entire country.

In the current analysis, the primary outcome measure focused on all-cause mortality.

### 2.6. Definition of Endpoints

The principal objective of this investigation was to evaluate the influence of low bone mineral density (BMD) on the occurrence of all-cause mortality subsequent to TAVR.

### 2.7. Statistical Analysis

Categorical variables are reported as frequencies and percentages. Continuous variables are presented as mean values ± standard deviation (SD). Restricted cubic spline analysis was performed. Kaplan–Meier curves were used for survival estimation. Univariable and multivariable Cox proportional hazard models were used to calculate hazard ratios (HRs), 95% confidence intervals (CIs), and *p*-values. All *p*-values were two-sided, and a *p*-value < 0.05 was considered significant for all tests. All statistical analyses were performed with SPSS and Stata.

## 3. Results

### 3.1. Baseline Clinical Characteristics

We included a total of 770 patients (54.0% male) with a mean age of 80.7 ± 6.7 years.

Regarding the excluded patients, a detailed comparative analysis between included and excluded patients is presented in Table S1 of the Supplementary. Table 1 lists the baseline characteristics of the entire cohort, stratified by a threshold of 200HU, derived as stated below. The average HU was 147.5 ± 75.4 (entire cohort). Comparing low BMD with a threshold of <200 HU, *n* = 614 (79.7%) patients had <200 HU, and *n* = 156 (20.3%) had ≥200 HU.

Low BMD was more likely in elderly patients with an age of 81.0 (6.5) years in HU < 200 and an age of 79.5 (7.6) years in HU ≥ 200 with a *p* = 0.009. Further, men had also significantly lower BMD with 52.0% in HU <200 and with HU ≥ 200 62.2% with *p* = 0.022 (Table 1).

**Figure 2:**
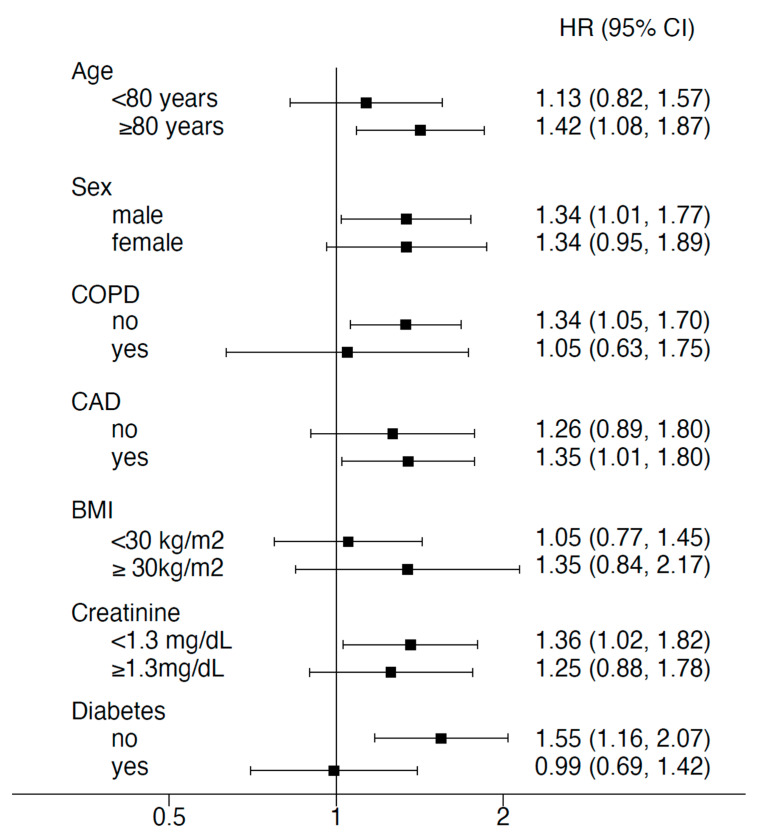
Forest plot showing the association of parameters with low BMD < 200 HU. COPD = chronic pulmonary disease; CAD = coronary artery disease; BMI = body mass index.

No differences were observed for COPD with 8.5% in HU < 200 and 7.7% in HU ≥ 200 (*p* = 0.076), creatinine with 1.3 (0.8) in HU < 200 and 1.3 (0.8) in HU ≥ 200 with *p* = 0.94, CAD with 52.5% in HU < 200 and 48.3 in HU ≥ 200 with *p* = 0.037, smoking with 3.1% in HU < 200 and 2.8HU ≥ 200 with *p* = 0.87, or BMI with 27.5 (5.4) in HU < 200 and 28.6 (5.4) HU ≥ 200 with *p* = 0.11 (Figure 2). The association of parameters with low BMD are shown in Figure 2.

### 3.2. Survival

During a mean follow-up of 28.1 ± 13.4 months, a total of 187 (24.4%) patients died.

Upon crude Cox regression analysis, every 100 HU decrease was associated with a 32% increase in risk for death (HR 1.32 [95%CI: 1.068–1.65)], *p* = 0.010).

**Figure 3:**
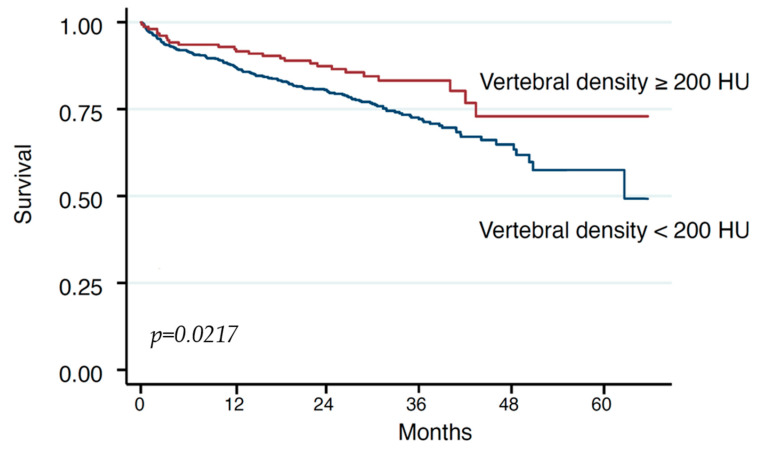
Kaplan–Meier estimates of survival in low BMD after TAVR.

Upon restricted cubic spline assessment, we observed a stark increase in risk for death when using a cut-off of 200 HU with lower HU values indicating higher risk (Figure 3).

When adjusted for age, sex, and NT-proBNP values, BMD remained significantly associated with death (adj. HR per 100HU decrease: 1.27 [1.01–1.59], *p* = 0.041).

In addition, we conducted individual univariable analyses for each variable, pinpointing those demonstrating significance for mortality (Supplementary Table S2). Subsequently, the relevant significant variables were integrated into the multivariable model, unveiling a notable decrease in survival rates linked to low bone mineral density (BMD). The variables included in the model encompassed BMD, COPD, NT BNP, diabetes, CAD, cancer, AF, and creatinine, all of which exhibited significance. Additionally, sex and age were included as acknowledged predictors for impaired BMD. Notably, after the univariate analyses, low bone mineral density retained significance with a *p*-value of 0.043.

## 4. Discussion

Our study findings reveal a significant association between low bone mineral density (BMD) measured on pre-TAVR computed tomography (CT) scans and reduced survival following TAVR. We observed a notable increase in the risk of death with decreasing Hounsfield unit (HU) values, with lower HU values indicating higher risk. Specifically, every 100 HU decrease was associated with a 32% increase in the risk of death. These findings suggest that BMD measurements on TAVR-CT scans could serve as a valuable tool for comprehensive frailty assessment and improved risk prediction in patients undergoing TAVR.

Over the past two decades, the adoption of TAVR has surged due to significant advancements in safety and efficacy. While initially reserved for high-surgical-risk patients with severe aortic valve stenosis, its indications have expanded to include intermediate and selected low-surgical-risk patients, thereby broadening its therapeutic reach. Notably, TAVR has emerged as a crucial treatment option for patients deemed unsuitable for surgical intervention. Given this paradigm shift, a thorough frailty assessment has become indispensable and is routinely conducted for each patient undergoing TAVR.

In addition to established risk assessment tools such as the Society of Thoracic Surgeons (STS) and the European System for Cardiac Operative Risk Evaluation (EuroSCORE)-II scores, recent studies have explored further tools for objective frailty risk estimation. Notably, body composition parameters, including muscle mass and fat distribution, have garnered significant attention.

Here specifically, the impact of fat and muscle mass distribution was studied intensively. Regarding muscle mass, multiple studies observed that sarcopenia is related to subsequent frailty and instability in the elderly population [35]. Measuring sarcopenia by using a CT scan or ultrasound has proved effective in practice [10,11,12,13,14,15,16]. Also, psoas muscle area and subcutaneous fat were analyzed and showed to be predictors for poor outcomes after TAVR [17,18,19,20]. Moreover, in a prior inquiry conducted by Demirel et al., a volumetric examination employing pre-TAVR computed tomography (CT) scans centered on a solitary axial image obtained at the middle third lumbar vertebra (L3) level, primarily aimed at delineating the attributes of visceral adipose tissue (VAT) and total muscle area (TMA). This inquiry underscored the association between diminished TMA and heightened 5-year all-cause mortality, thus introducing an auxiliary criterion for the assessment of frailty. Additionally, the influence of reduced TMA on 5-year all-cause mortality displayed sex specificity, notably revealing a protective effect linked to elevated TMA levels among male subjects. In contrast, neither appendicular muscle area (PMA) nor VAT demonstrated a significant correlation with escalated 5-year mortality rates. Moreover, the amalgamation of TMA and VAT did not augment the prognostic potency for predicting diminished survival rates [7]. 

Another critical body composition parameter implicated in frailty across multiple conditions is low BMD. However, sparse data exist regarding its impact on TAVR outcomes. In noncardiac diseases, low BMD has been independently associated with poor prognosis, particularly in cancer patients [36,37]. Lower BMD was also significantly associated with increased cardiovascular risk [21,22]. The main complications as a consequence of low BMD are OP and osteoporotic fracture, which leads to frailty in older patients [22]. Parameters associated with osteoporotic fractures include, among others, low BMD, age, sex, hormonal factors, specific medication, cigarette smoking, low physical activity, race, or small body size [29,38].

Our study corroborates previous findings, demonstrating that increasing age and male sex are associated with low BMD, with previous studies also confirming the increased mortality risk in men with low BMD. Increased risk of mortality in men with low BMD was proven in previous studies [23]. Further, previous studies also observed that male osteoporosis is underscreened, underdiagnosed, and undertreated, both in primary and secondary prevention of fragility fractures [39]. Concerning COPD, previous studies observed that these patients have a high prevalence of osteoporosis and a high risk of fracture [40,41]. For our cohort, no significant difference was observed. This might be due to the different impacts of each predictor on developing OP.

Opportunistic BMD measurements in abdominal and thoracic CT scans obtained for other clinical indications have previously been performed to identify individuals at risk of low BMD and subsequent fractures [30,31,32]. Thoracic BMD was measured on routine cardiac CT to identify individuals who have low BMD and a greater fracture rate [33]. In the present study, we observed that BMD measurements can also be performed in pre-TAVR-CT at the thoracal spine with a significant impact on long-term survival.

In summary, our findings underscore the significant association between low BMD and reduced survival following TAVR. The opportunistic measurement of BMD in TAVR workup CT scans holds promise as a tool for comprehensive frailty assessment and improved risk prediction in TAVR patients, thereby facilitating more informed clinical decision making and optimizing patient outcomes in the era of transcatheter interventions for aortic valve replacement.

## 5. Study Limitations and Future Directions

One of the primary limitations of our study stems from the demographic characteristics of the study population, which predominantly consists of elderly individuals affected by both severe AS and OP. Given the inherent predisposition of the elderly to frailty and multimorbidity, the complexity of these comorbid conditions may confound the interpretation of our findings, particularly regarding the distribution of BMD values.

Moreover, the diverse treatment regimens employed for AS and OP among our study participants introduce additional complexities. Variations in medication usage, such as corticosteroids known to increase osteoporosis risk, or osteoporosis therapies like calcium, vitamin D, or bisphosphonates may impact BMD levels and thereby influence the observed associations with TAVR outcomes. The heterogeneous medication profiles within our cohort present a potential source of variability in BMD measurements and underscore the need for cautious interpretation of our results.

Future prospective studies are warranted to elucidate the causal relationships between BMD, osteoporosis treatment modalities, and TAVR outcomes. Specifically, investigating the impact of post-detection osteoporosis management on TAVR outcomes may yield valuable insights into strategies for optimizing patient care and enhancing procedural outcomes. Longitudinal studies tracking changes in BMD over time and correlating these changes with clinical outcomes post-TAVR could provide valuable insights into the dynamic nature of bone health and its impact on TAVR prognosis.

Furthermore, despite meticulous planning and execution, we encountered inherent challenges that influenced the comprehensiveness of our data collection process. Notably, due to the decentralized nature of data acquisition, particularly in cases conducted at referring centers, we faced difficulties in obtaining complete sets of CT scans for measurements. As a consequence, a subset of cases lacked the requisite CT scans necessary for inclusion in our analysis, thereby potentially introducing bias into our results.

Moreover, our study was predominantly reliant on contrast-enhanced CT scans for bone mineral density (BMD) measurements, as routine non-enhanced CT scans were not uniformly performed across our patient cohort. Furthermore, the exclusion of cases lacking non-enhanced CT scans raises concerns regarding the generalizability of our findings to a broader patient population.

Additionally, the decision to exclude cases lacking non-enhanced CT scans was motivated by the current lack of consensus regarding the potential impact of contrast agents on BMD measurements. While the existing literature has explored the comparison between enhanced and non-enhanced CT scans concerning BMD measurements, the applicability of such findings within the specific context of TAVR CT scan protocols remains uncertain.

Furthermore, while we acknowledge the established association between low BMD and various comorbidities, including cardiovascular diseases, our study did not find a significant difference in BMD concerning other comorbidities. This contrasts with findings from previous seminal studies, such as meta-analyses, which have demonstrated a clear association between low BMD and conditions like coronary artery disease (CAD) [42]. However, the interpretation of such associations may be confounded by common risk factors, given the inherent heterogeneity across study populations and methodologies. Thus, similar confounding factors may underlie the outcomes observed in our study, warranting cautious interpretation of our results in the broader context of bone health and its implications for cardiovascular health.

Additionally, exploring novel imaging modalities or biomarkers that offer more comprehensive assessments of bone quality and fracture risk beyond traditional BMD measurements may enhance risk stratification and prognostication in TAVR patients. Integration of advanced imaging techniques such as magnetic resonance imaging (MRI) to assess bone microarchitecture and strength could provide valuable insights into fracture risk prediction and guide personalized treatment approaches.

Furthermore, investigating the role of lifestyle interventions, such as exercise regimens or nutritional supplementation, in preserving bone health and reducing fracture risk in TAVR patients represents a promising avenue for future research. By incorporating multidisciplinary approaches that address both cardiovascular and musculoskeletal health, future studies have the potential to optimize patient outcomes and improve the quality of life in TAVR recipients.

In conclusion, while our study contributes valuable insights to the existing literature on BMD assessment in TAVR patients, it is essential to acknowledge and address these inherent limitations in future investigations. By embracing multidisciplinary collaborations, leveraging advanced imaging technologies, and exploring innovative therapeutic strategies, we can advance our understanding of the complex interplay between osteoporosis, frailty, and TAVR outcomes, ultimately enhancing patient care and clinical outcomes in this vulnerable population.

## 6. Conclusions

In conclusion, our study underscores the critical role of BMD assessment in pre-TAVR evaluations. We found a significant correlation between low BMD, identified through pre-TAVR-CT scans, and diminished survival rates post-procedure. Specifically, a decrease of 100 Hounsfield units (HUs) in BMD correlated with a notable 32% increase in mortality risk. Integrating BMD measurements into pre-TAVR assessments provides valuable insights for risk prediction and personalized intervention strategies. This approach enables clinicians to refine risk stratification protocols and tailor interventions to optimize therapeutic outcomes. By addressing bone health early in the TAVR evaluation process, clinicians can mitigate the adverse effects of low BMD and improve long-term survival prospects for TAVR patients. Ultimately, our findings underscore the evolving role of BMD assessment in transcatheter valve interventions, offering a promising avenue for enhanced patient care and improved outcomes in TAVR procedures.

## Figures and Tables

**Table 1:** Baseline characteristics.

	Total	HU < 200	HU ≥ 200	*p*-Value
	*n* = 770	*n* = 614	*n* = 156	
**Demographics**				
Age	80.7 (6.7)	81.0 (6.5)	79.5 (7.6)	0.009
Sex (male)	54.0%	52.0%	62.2%	0.022
Height	167.3 (9.7)	167.1 (9.8)	168.1 (9.5)	0.38
Weight	77.7 (16.9)	77.0 (16.8)	80.9 (17.1)	0.055
BMI kg/m^2^	27.7 (5.4)	27.5 (5.4)	28.6 (5.4)	0.11
Euro score II	4.8 (3.5)	4.8 (3.8)	4.7 (1.6)	0.79
LVEF (%)	56.7 (12.9)	57.0 (13.1)	55.5 (11.8)	0.21
**Comorbidities**				
AF before TAVR	22.4%	21.2%	27.6%	0.33
Diabetes	25.3%	24.3%	29.4%	0.21
Hypertension	57.4%	58.4%	53.1%	0.25
Smoking	3.0%	3.1%	2.8%	0.87
PAD	6.8%	6.6%	7.7%	0.65
CVD	1.4%	1.7%	0.0%	0.12
CHF	19.0%	19.9%	15.4%	0.22
CAD	51.6%	52.5%	48.3%	0.37
CVI	10.8%	11.4%	8.4%	0.30
CABG	5.9%	5.8%	6.3%	0.81
COPD	8.3%	8.5%	7.7%	0.76
Cancer	14.5%	15.4%	10.5%	0.13
**Laboratory findings**				
Creatinin mg/dL	1.3 (0.8)	1.3 (0.8)	1.3 (0.8)	0.94
Triglycerides mg/dL	110.4 (57.1)	109.5 (58.6)	114.0 (50.6)	0.41
Cholesterol mg/dL	144.1 (42.6)	144.4 (43.3)	142.7 (39.5)	0.66
HDL-C mg/dL	55.1 (16.6)	55.7 (17.1)	52.6 (14.3)	0.061
LDL-C mg/dL	74.6 (34.7)	75.6 (35.3)	70.3 (31.9)	0.14
Bilirubin mg/dL	0.7 (0.5)	0.6 (0.5)	0.7 (0.4)	0.59
GOT U/L	30.0 (72.4)	30.8 (80.1)	26.6 (18.3)	0.53
GPT U/L	26.4 (53.4)	27.3 (59.0)	22.9 (15.9)	0.37
Hemoglobin g/dL	12.1 (1.9)	12.0 (2.0)	12.3 (1.9)	0.14
INR	1.1 (0.2)	1.1 (0.2)	1.1 (0.2)	0.70
Thrombocytes G/L	218.1 (74.6)	218.3 (75.7)	217.0 (69.8)	0.85
HbA1c %	6.0 (0.9)	5.9 (0.9)	6.1 (0.9)	0.12
NTproBNP pg/mL	4286.8 (6158.1)	4407.9 (6317.0)	3773.4 (5425.0)	0.29
**CT bone density**				
Average HU in thoracal vertebra	147.5 (75.4)	120.0 (44.8)	255.8 (73.8)	<0.001

AF = atrial fibrillation; PAD = peripheral artery disease; CVD = cerebrovascular disease; CHF = chronic heart failure; CAD = coronary artery disease; CVI = cerebrovascular insult; CABG = coronary artery bypass grafting; COPD = chronic pulmonary disease; GOT = serum glutamic oxaloacetic transaminase; GPT = serum glutamic pyruvic transaminase; HDL-C = high-density lipoprotein-cholesterol; LDL-C = low-density lipoprotein-cholesterol; INR = international normalized ratio; HbA1c = glycated hemoglobin; NTproBNP = N-terminal pro-B-type natriuretic peptide.

## Data Availability

The data underlying this article are available in the TAVR Register of Vienna and will be shared upon reasonable request to the corresponding author.

## Supplementary Materials

The following supporting information can be downloaded at: https://www.mdpi.com/article/10.3390/jcm13092698/s1. Table S1: A descriptive analysis comparing included and excluded patients. Table S2: Univariate Analysis.

## Abbreviations

TAVR	Transcatheter Aortic Valve Replacement
SAVR	Surgical Aortic Valve Replacement
BMD	Bone Mineral Density
OP	Osteoporosis
AS	Aortic Valve Stenosis
CT	Computed Tomography
