# Supplementary material for: Low Bone Mineral Density on Computed Tomography: Association with Poor Survival after Transcatheter Aortic Valve Replacement
Source: J Clin Med. 2024 May 3;13(9):2698. doi: 10.3390/jcm13092698 (PMC11084390; doi:10.3390/jcm13092698)
Supplement: Supplementary file 1 [file jcm-13-02698-s001.zip › jcm-2974440-SM.pdf]

Supplementary Materials

Table S1. A descriptive analysis comparing included and excluded patients.

	Excluded<br>N=452	Included<br>N=770	p-value
Age	81.1 (6.97)	80.7 (6.72)	0.387
Sex	0.49 (0.50)	0.54 (0.50)	0.077
CT bone density	NA	147 (75.4)	
Diabetes	0.23 (0.42)	0.25 (0.43)	0.367
Hypertension	0.58 (0.49)	0.57 (0.49)	0.927
Smoking	0.02 (0.15)	0.03 (0.17)	0.503
PAD	0.05 (0.23)	0.07 (0.25)	0.355
CAD	0.03 (0.16)	0.01 (0.12)	0.180
Heart Failure	0.19 (0.39)	0.19 (0.39)	0.991
CAD	0.48 (0.50)	0.52 (0.50)	0.192
CVI	0.12 (0.32)	0.11 (0.31)	0.655
CABG	0.06 (0.23)	0.06 (0.24)	0.913
COPD	0.12 (0.32)	0.08 (0.28)	0.064
AF	0.30 (0.46)	0.27 (0.44)	0.244
Cancer	0.15 (0.36)	0.14 (0.35)	0.708
Creatinin mg/dl	1.29 (0.85)	1.27 (0.81)	0.679
Triglycerides mg/dl	114 (55.3)	110 (57.1)	0.259
Cholesterol mg/dl	147 (44.6)	144 (42.6)	0.272
Bilirubin mg/dl	0.63 (0.39)	0.65 (0.46)	0.400
GOT U/L	27.6 (23.1)	30.0 (72.4)	0.415
GPT U/L	24.2 (23.2)	26.4 (53.4)	0.329
NTproBNP pg/ml	4962 (7048)	4287 (6158)	0.129
Hemoglobin g/dl	11.7 (1.85)	12.1 (1.94)	0.005
LVEF %	55.9 (13.6)	56.7 (12.9)	0.373
GFR	58.1 (24.9)	59.7 (23.8)	0.430
EuroScore_II	4.42 (0.96)	4.77 (3.54)	0.052
AF= Atrial fibrillation; PAD= peripheral artery disease; CVD= Cerebrovascular disease; CHF= chronic heart failure; CAD= Coronary artery disease; CVI= Cerebrovascular insult; CABG= Coronary artery bypass grafting; COPD= Chronic pulmonary disease; GOT= serum glutamic oxaloacetic transaminase; GPT= serum glutamic pyruvic transaminase; NTproBNP= N-terminal pro–B-type natriuretic peptide;			

Table S2. Univariate Analysis.

	95 % CI	p value
Age	1.01 [0.99;1.04]	0,196
Sex	1.14 [0.86;1.53]	0,362
Height	1.02 [1.00;1.04]	0,104
Weight	1.01 [0.99;1.02]	0,347

BMI kg/m2	1.00 [0.97;1.04]	0,915
Euro_score_II	1.04 [1.01;1.06]	0,007
LVEF (%)	0.99 [0.97;1.00]	0,016
Co-morbities		
AF before TAVR	1.70 [1.26;2.31]	0,001
Diabetes	1.49 [1.09;2.03]	0,013
Hypertension	0.97 [0.72;1.31]	0,853
Smoking	2.18 [1.19;4.02]	0,012
PAD	2.03 [1.29;3.21]	0,002
CVD	1.75 [0.65;4.71]	0,27
CHF	1.77 [1.28;2.45]	0,001
CAD	1.37 [1.01;1.85]	0,04
CVI	1.34 [0.87;2.06]	0,184
CABG	2.07 [1.27;3.37]	0,004
COPD	1.94 [1.28;2.94]	0,002
AF	1.70 [1.26;2.31]	0,001
Cancer	1.65 [1.16;2.37]	0,006
CT bone density		
CT bone density	1.32 [1.068;1.65]	0,010
AF= Atrial fibrillation; PAD= peripheral artery disease; CVD= Cerebrovascular disease; CHF= chronic heart failure; CAD= Coronary artery disease; CVI= Cerebrovascular insult; CABG= Coronary artery bypass grafting; COPD= Chronic pulmonary disease		
